# Cooling effect of urban forests on the urban heat island in Seoul, South Korea

**DOI:** 10.1371/journal.pone.0288774

**Published:** 2023-07-21

**Authors:** ByungMook Hwang, Hong-Duck Sou, Jeong-Hak Oh, Chan-Ryul Park

**Affiliations:** 1 Urban Forests Division, National Institute of Forest Science, Seoul, Korea; 2 Department of Forest Sciences, Kookmin University, Seoul, Korea; Southwest Jiaotong University, CHINA

## Abstract

Air pollution and climate change amplify the urban heat island (UHI) effect, which has an adverse effect on human health. Urban forests (UFs) are important to reduce the UHI effect; however, the quantitative effect of UFs on UHI, relative to time and space, has not yet been investigated. In this study, we aimed to quantitatively measure the actual thermal environment in UFs. To this end, temperature and humidity loggers were installed in 17 UFs in Seoul for a year and analyzed according to vegetation characteristics and accessibility. The urban forests and park showed consistent temperature reduction, whereas the lawn showed higher temperature reduction effects during autumn-winter. The traffic island showed lower annual temperature reduction effect than other UFs. From spring to autumn, mixed and broadleaved forests showed better temperature reduction effect than coniferous forests. The temperature in UFs decreased by approximately 1.9°C over ~3 km from the traffic island near the city to the forest. This study revealed the difference in the cooling effect according to the type and location of UF and the vegetation structure. The functional characteristics of plants and the UF that reflects them can help reduce the negative impact of climate warming and UHI on human health.

## Introduction

Migration of people from rural to urban areas has increased in recent decades. This has resulted in more than half the world’s population living in cities and towns since 2008. This proportion is expected to rise to 70% by 2050. As cities expand, their natural landscapes are reconstructed and altered, creating a microclimate that differs from that of the surrounding countryside in terms of temperature, rainfall, and wind [1].

Land use patterns, particularly development intensity and vegetation, influence ambient temperature of a region through differential rates of energy storage, which are released with contributions from anthropogenic heat sources, as reported by Ward and Grimmond [2]. Plant and soil moisture content are important drivers of energy distribution. Energy absorbed from the surface and released as sensible heat increases the temperature near the surface, whereas energy used to evaporate water through the ground or vegetation does not affect the surface temperature. Nighttime cooling rates in densely built areas are lower than those in open vegetated areas because of building configurations that prevent daytime heat absorption and release, heat generation, ventilation, and radiative cooling [3].

Green infrastructure can be defined as all natural, semi-natural, and artificial networks of multifunctional ecosystems within, around, and between urban areas at all spatial scales. It includes a wide range of ecosystem services (ESS) that provide climate regulation as an interconnected network of natural and semi-natural features including green spaces, trees, water bodies, green roofs, and vertical greenery [4–12]. Urban areas occupy 4% of the world’s land area and increasingly include plantings based on the ESS that UFs can provide [12–14]. ESS commonly refers to the following functions and benefits of urban forests: 1) emotional and psychological services, 2) physiological services, 3) monetary services, 4) hydrological services, 5) thermal services, 6) air quality services, 7) synurbization services, and 8) population and community services [12]. UFs comprise all trees in urban areas, including individual street trees, park tree clusters, and peri-urban forests extended to outer metropolitan areas to establish a network. Types of UFs include city parks, pocket parks, trees on streets, gardens with trees, public squares with trees, and other green spaces with trees, such as schools, riparian corridors, and rooftops.

Urban overcrowding resulting from urbanization has simultaneously accelerated urban development and reduced green areas. As the number of man-made structures increases, the area of impermeable pavement on the ground, called gray infrastructure, increases. Moreover, moisture conditions become poor, and radiant heat and heat storage from man-made structures accumulate between high-rise buildings, exacerbating the urban heat island phenomenon. Urban forests (UFs) on the surface block heat energy from the sun through the shade effect and lower its internal temperature by vaporizing moisture through evapotranspiration. The ground surface, which has superior water permeability compared to gray infrastructure, produces high humidity. UFs block hot sunlight during the day and has a lower temperature range at night compared to gray infrastructure because of the radiative cooling effect.

Research on urban heat island (UHI) mitigation using the temperature reduction effect of UFs is actively being conducted. Recently, studies on urban climate analysis and thermal environment evaluation have been attempted through various simulation programs (such as Envi-met and RayMan, among others) and analysis tools such as GIS that can analyze urban spatial characteristics [15–20]. As remote sensing techniques become more advanced, temperatures of large gray infrastructure can be easily obtained with the advantage of stably collecting high-resolution data.

A difference in the land surface temperature (LST) and the temperature through satellite has been observed [21]. As most studies that measure temperature are short-term, either measured throughout the year or during summer when hot days are concentrated, studies on the temperature reduction effect of UFs conducted for only one year have insufficient data. In the case of satellite imagery, mid- to long-term LST is analyzed; however, continuous temperature observation is difficult because of the limitation of the shooting cycle, and it is difficult to accurately observe the temperature of UFs in small cities. Therefore, analyzing the difference in the mid- to long-term temperature reduction effect of adjacent UFs through continuous temperature measurement for one year is necessary.

Surface temperature measured using artificial satellites may differ from the actual temperature perceived by citizens in UFs and gray infrastructure. In summer, owing to the development of the crown layer, confirming the temperature difference between urban forests and gray infrastructure over a 24-hour period becomes more difficult. Currently, research is mainly conducted on urban climates such as UHI and wind roads rather than on the effects of the thermal environment on humans. In addition, research on small spaces has only considered the physical characteristics of the space, and the interrelationship between thermal environmental factors that affect human sensation and behaviors has been overlooked [22]. Therefore, there is a need for systematic research and evaluation of small-scale spaces to establish a management plan suitable for local characteristics and to improve the design of practical urban spaces.

Most research in the field of thermal environment is based on urban-scale approaches. However, proposing a specific use plan for the improvement of the UF thermal environment when considering actual UF users is difficult. Therefore, quantitative evaluation of small UFs, such as traffic islands (Ti) and urban neighborhood parks, which are easily accessible to citizens living in cities, is necessary. Furthermore, guidelines to systematically manage and improve the thermal environment of UFs should be established.

**Fig 1:**
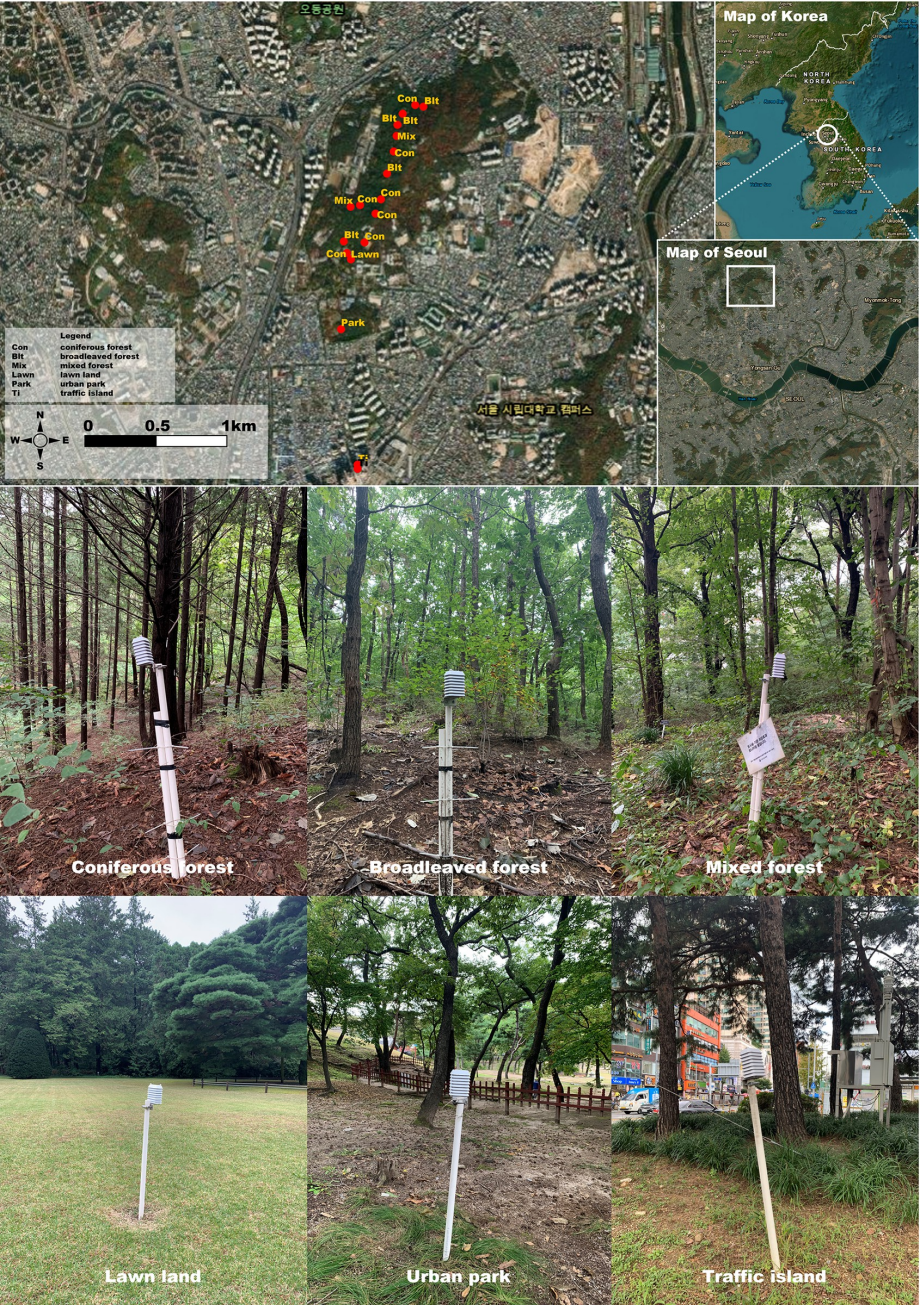
Study area and location of the study sites. Monitoring was performed at 17 sites, located in six different types of urban forests in Seoul, South Korea. The map was generated using QGIS 3.16.16 (http://www.qgis.org/).

Since urbanization and climate change have a great impact on quality of life, various studies on UHIs are being conducted mainly in Seoul and East Asia, which are undergoing rapid urbanization. The cooling effect of UHIs has been analyzed based on urban climate and topography, surface characteristics, and vegetation data [23–26]. Although various studies have been conducted, studies that directly quantify the cooling effect on various UFs in urban areas are still needed. Therefore, in this study, the cooling effect of UFs in the city center of Seoul was investigated and the degree of cooling was quantified (Fig 1). To this end, we tried to analyze the impact of the temperature reduction effect by analyzing the temperature change through long-term monitoring of various types of stand structures of UFs, which are expected to have a direct impact on urban temperature reduction.

## Results

### 1) Stand structure characteristics of study sites

The main tree species at each study area were *Pinus densiflora* (Ti); *Quercus acutissima* (Park); *Pinus densiflora* (Con); *Quercus mongolica* and *Quercus palustris* (Blt); and *Pinus densiflora*, *Abies holophylla* and *Acer palmatum* (Mix) (Table 1).

**Table 1:** Stand structure characteristics of five urban forest types near the Hongneung experimental forest, Seoul. Values are shown as the mean (n = 4–13) ± standard deviation.

Study area	*Ti*	*Park*	Con	*Blt*	*Mix*
**Main species**	*Pinus densiflora*	*Quercus acutissima*	*Pinus densiflora*	*Quercus mongolica* *Quercus palustris*	*Pinus densiflora Abies holophylla Acer palmatum*
**DBH (cm)**	23.8±1.3	38.8±7.7	12.3±2.5	18±7.2	25.9±13.2
**Height (m)**	10.2±2.2	15.3±1	10.4±0.8	14.1±4.6	11.3±13.2
**Crown depth (m)**	3.8±2.6	8.8±0.7	2.9±0.6	9.8±5.0	6.2±3.0
**Clear length (m)**	6.1±2.0	6.6±0.6	7.5±0.1	4.7±3.4	4.9±3.8
**Basal area (m** ^**2**^ **)**	0.2	5	0.2	0.3	0.5
**Crown diameter (m)**	2.8±0.4	5.2±1.3	1.4±0.5	1.9±0.8	3.5±1.6
**Stem volume (m** ^**3**^ **)**	2.24	7.3	1.7	4.84	7.3
**Crown projection area (m** ^**2**^ **)**	128.7	349.7	86.5	128.2	366.3
**Crown volume (m** ^**3**^ **)**	184.88	1977.47	180.2	804.08	1862.3
**Forestland area (m** ^**2**^ **)**	100				

Ti, traffic island; Park: urban park; Con: coniferous forest; Blt: broadleaved forest; Mix: mixed forest; DBH: diameter at breast height.

### 2) Seasonal and diurnal cooling effect of urban forests

**Fig 2:**
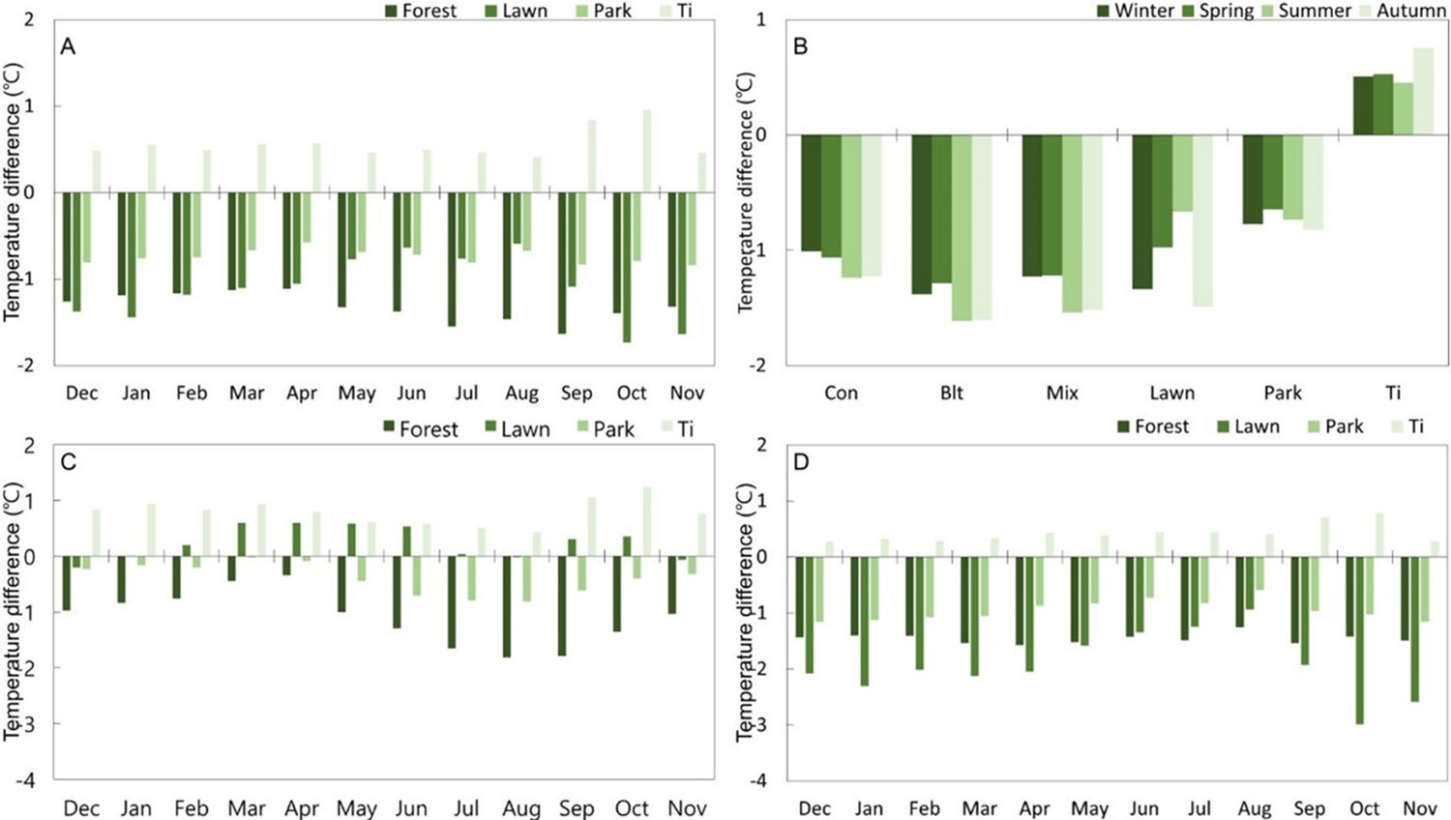
Seasonal differences in cooling effects of urban forests. (A, B) Seasonal differences in cooling effects of different types of urban forests. (B) Winter: Dec–Feb; Spring: Mar–May; Summer: Jun–Aug; Autumn: Sep–Nov. (C, D) Seasonal differences in cooling effects of different types of urban forests during day/nighttime. (C) Day: 06:00–19:00; (D) Night: 20:00–05:00.

The urban forests and park showed consistent temperature reduction throughout the year. The temperature of the forests was 1.3°C and 1.1°C lower than that of gray infrastructure in winter and spring, respectively. The temperature difference was maintained throughout summer (July to September) but decreased thereafter. The park showed a pattern similar to that of the forest. The lawn showed a similar level of temperature reduction effect to that of the park in summer, but the difference nearly doubled in autumn and winter. Unlike the park, which exhibited a constant temperature reduction effect throughout the year, the lawn showed a relatively high temperature reduction effect in Oct–Jan compared to forests and the park (Fig 2). In addition, the Ti showed a lower annual temperature reduction effect than the other UFs (Fig 2).

The daytime temperature reduction in the forest was similar to the pattern in Fig 2; however, the range of change was large. After winter (−1.0°C), temperature reduction decreased to 0.3°C in spring, then increased rapidly from May, and improved to −1.8°C in August. Thereafter, as the leaves fell, the daytime temperature reduction effect decreased. The park also showed a similar weekly temperature distribution. The shading effect of the tree canopy contributes to a cooler environment on the surface of the plants [27]. The daytime temperature of the lawn was higher than that of the gray infrastructure in spring and autumn, when the daily temperature difference was large, and there was no temperature reduction effect in winter and summer, when the temperature change was small. The Ti maintained an average monthly temperature 0.4–1.0°C higher than that of the gray infrastructure throughout the four seasons. The temperature gradually decreased from early spring to late summer and increased in the autumn (Fig 2).

UFs showed a certain level of nighttime temperature reduction throughout the year, except for the lawn. Forests showed a temperature reduction of −1.3 to −1.6°C throughout the year, and the park showed a reduction of −0.6 to 1.2°C. All the UFs showed the lowest temperature reduction effect in August. The lawn showed the greatest nighttime temperature reduction effect among UFs, except in the summer, and considerable differences in temperature reduction were observed by month. In particular, the temperature reduction effect in autumn (−1.9 to −3.0°C) was remarkably increased, which is contrary its daytime temperature reduction effect (Fig 2).

**Fig 3:**
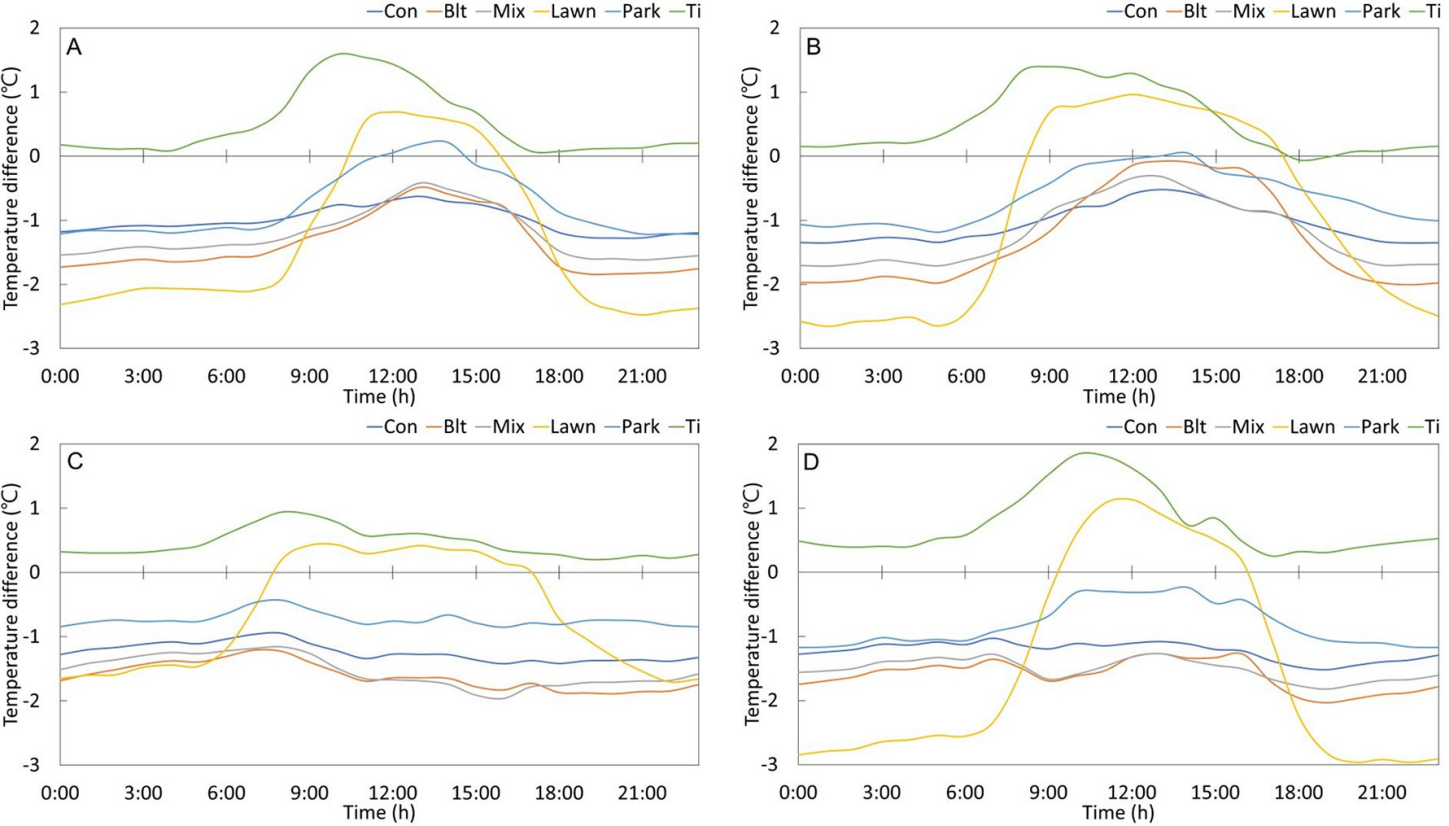
Diurnal differences in the cooling effects of urban forests for each season. (A) Winter: Dec–Feb; (B) Spring: Mar–May; (C) Summer: Jun–Aug; (D) Autumn: Sep–Nov.

The diurnal temperature difference was the maximum for the lawn in all seasons. The seasonal characteristics of the large diurnal temperature difference revealed that it was high in autumn and spring, and lowest in the summer when the temperature in all types of UFs was high. Daytime and nighttime average temperatures showed different distributions. Regardless of the season, during the daytime, the Ti, Automatic Weather System (AWS), and lawn formed a hot zone, while at night, the hot zone was formed by the Ti, AWS, and park. The coniferous, broadleaved, and mixed forests formed a low-temperature zone during the daytime; while the lawn exhibited the lowest average temperature at night, except in summer (Fig 3).

### 3) Cooling effect in urban forests by forest type

**Fig 4:**
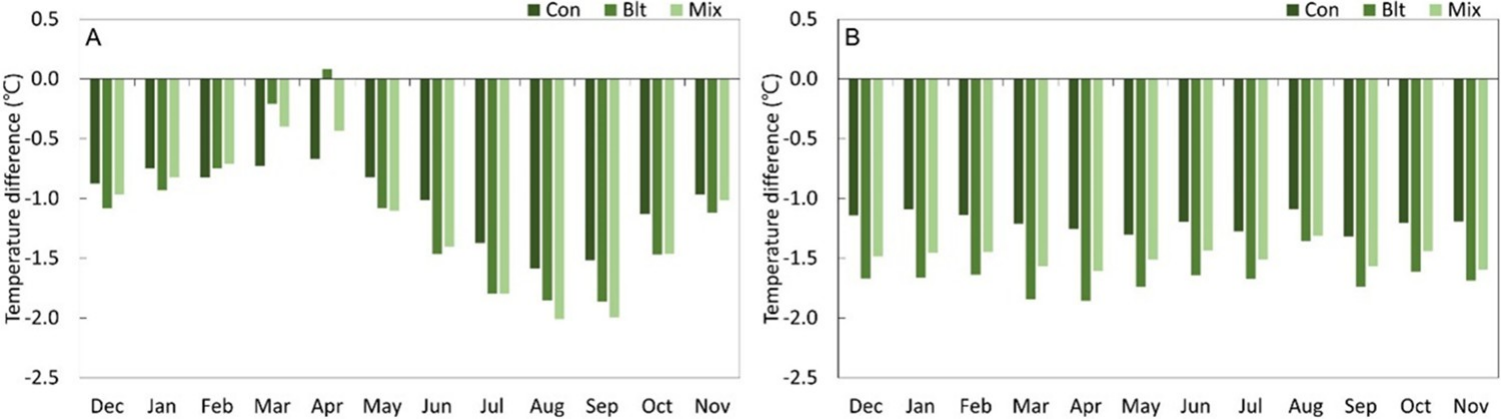
Seasonal differences in day and night cooling effects of three different urban forest types. (A) Day: 06:00–19:00; (B) Night: 20:00–05:00.

Temperature reduction was confirmed by classifying forests into coniferous, broadleaved, and mixed. The temperature reduction in broadleaved and mixed forest, which start to thrive in spring, decreased compared to other seasons, and broadleaved forests recorded a higher temperature (0.1°C) than the AWS. From late spring (May), the ability of broadleaved and mixed forests to reduce temperature became superior to that of coniferous forests, and this phenomenon continued until autumn. In particular, the ability of mixed forests to reduce the temperature in summer was superior to that of the broadleaved forest. At night, a constant temperature reduction was maintained regardless of the season. The temperature reduction in the different forest types is found in the following order: broadleaved forest > mixed forest > coniferous forest (Fig 4).

The cooling effect in UFs varied by forest type when they were compared for one hour. Coniferous forests showed a temperature reduction of 1.1±0.5°C throughout the year, and −1.2±0.5°C even in summer. The temperature variability in the summer was found to be similar to that observed in other seasons. In contrast, the temperature variability in broadleaved and mixed forests was −1.5±0.8°C and −1.4±0.7°C, respectively, which was superior to that observed in coniferous forests. The range of temperature variability in broadleaved forests (−1.3±1.0°C) and mixed forests (−1.2±0.7°C) in spring was larger than that in autumn. The summer mitigation fluctuations did not show a substantial difference. During this time, solar radiation and internal temperature increase due to seasonal changes.

### 4) Cooling effects of urban forests in the city center

**Fig 5:**
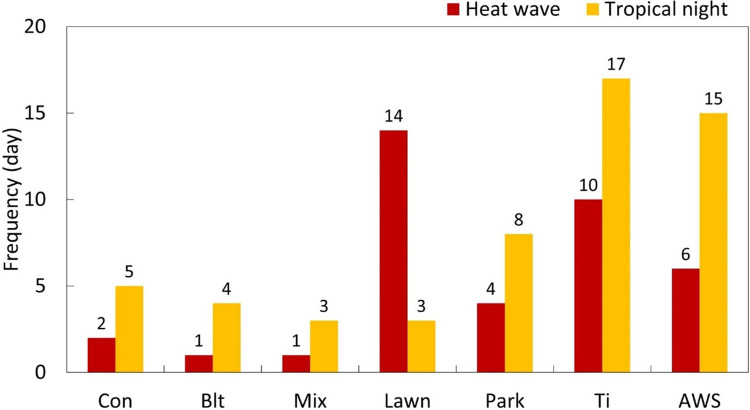
Cumulative frequency of abnormal temperature occurrences (heat wave and tropical night) at study sites during summer. Heat wave: daily maximum temperature exceeds 33°C; tropical night: minimum temperature exceeds 25°C after 6 p.m. and before 9 a.m.

Abnormal temperatures recorded in summer differed 5–10-fold (for hot days) and 3–5-fold (for tropical nights) when comparing the study areas. Hot days were frequently observed in the lawn (14 days), followed by Ti (10 days), AWS (6 days), park (4 days), coniferous forests (2 days), broadleaved forests (1 day), and mixed forests (1 day). Tropical nights were observed most frequently in the Ti and AWS, which are adjacent to gray infrastructure. Among the UFs, hot days most frequently occurred in the lawn, making it vulnerable to heat damage. However, the lawn showed the least occurrence of tropical nights, along with broadleaved and mixed forests (Fig 5).

**Fig 6:**
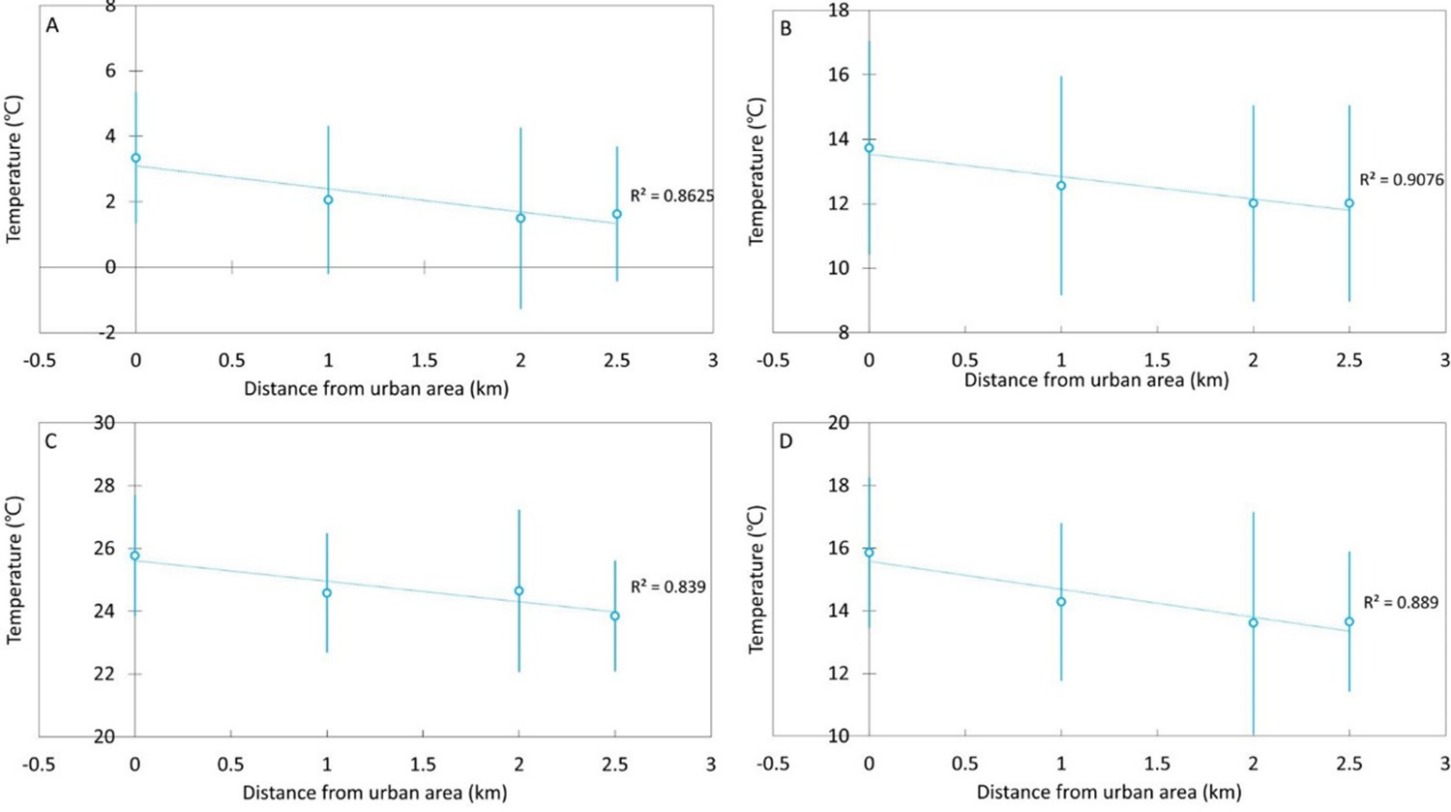
Effect of distance from the city center (gray infrastructure) on seasonal temperature in urban forests. (A) Winter: Dec–Feb; (B) Spring: Mar–May; (C) Summer: Jun–Aug; (D) Autumn: Sep–Nov.

Further analysis confirmed that the temperature decreases as the distance from gray infrastructure (city center) increases. This was observed not only in summer, when hot days and tropical nights occur, but in other seasons as well. The average temperature in the Ti was approximately 1.7–2.2°C higher than the average temperature in the urban forests (Fig 6).

## Discussion

Forests and parks with trees showed similar temperature reduction patterns, decreasing from winter to spring and increasing rapidly from spring to summer. The daytime temperature reduction effect decreased as leaves fell in the autumn. This is attributed to the shading effect by the tree canopy that contributes to a cooler environment [28–31]. Although lawns could possibly form a higher temperature zone than the Ti due to the absence of trees, this was not the case, as evidenced by the change in daily temperature (Fig 3). Owing to the lack of trees and consequently, shade, the lawn temperature increased rapidly after sunrise and decreased rapidly after sunset. For this reason, the highest daily temperature was recorded in the lawn, but the highest average daily temperature was recorded in the Ti. In the Ti, the average monthly temperature was 0.4–1.0°C higher than that in AWS throughout the four seasons. The temperature difference gradually decreased from early spring to late summer, and in autumn, a higher temperature range was observed in the Ti than in gray infrastructure (Fig 2). This is because the Ti, surrounded by gray infrastructure, formed a higher temperature area than the AWS, but due to the vegetation cover by trees (*Pinus densiflora*) and shrubs (*Rhododendron yedoense*) and a topographical cover by herbs, the insulation increased in spring, and temperature variation was caused by the shadow effect and evapotranspiration. Small UFs implemented near gray infrastructure, such as roads and Ti, also reduce temperature and are expected to serve as shelters on hot days.

The nighttime temperature reduction effect of UFs was observed at a constant level throughout the year except in lawns (Figs 2, 4). Gray infrastructure maintains a high temperature owing to sensible heat generated from human activities, buildings, and roads, while UFs maintain a constant level of temperature reduction throughout the year because of evapotranspiration. Although the nighttime cooling effect of UFs during summer is relatively low compared to that during winter, this difference is not significant. This is due to the higher heat energy received during the daytime in summer than that in winter. The high heat capacity of UFs allows the heat received during the daytime to slowly dissipate throughout the night, resulting in a relatively small temperature difference between UFs and built-up areas during summer nights [32–34]. The increase in daytime temperature reduction may be attributed to the shadow effect and evapotranspiration caused by high temperature, which are maximized in summer when the amount of insolation and leaf mass increases. The temperature reduction effect is evidently diminished as the temperature decreases. In spring and autumn, when the daily temperature fluctuates drastically, the lawn exhibits high temperatures during the day and low temperatures at night (Fig 3). In addition, since transpiration in shrubs and trees in UFs absorbs heat and plays an important role in lowering the surface temperature in the forest, the heat reduction effect of lower vegetation such as in lawns is pivotal when implementing UFs in the city. It is considered optimal to compose a multi-layered forest with a combination of trees, glue trees, and shrubs.

Coniferous and mixed forests that form a canopy inside the forest are less affected by seasonal variations. In contrast, a characteristic diurnal temperature difference was observed in broadleaved forests that have not yet developed a canopy (Fig 4). Considering the phenological characteristics of deciduous forests, during the leaf-out period (March and April), before the tree canopy has fully developed, direct sunlight increasingly penetrates the forest, leading to a decrease in shade and evapotranspiration effects and resulting in the observed temperature reduction pattern, which was distinct from that of coniferous and mixed forests. The development of the crown layer indicates that the ratio of transpiration and evapotranspiration is high [27, 35–38]. Canopy cover serves as a regulator of temperature inside the forest, and the amount and density of canopy cover, as measured by the leaf area index (LAI), is significantly positively correlated with the temperature reduction effect inside the forest [37]. Wei et al. [38] also found that canopy density had a significant positive correlation with both cooling and humidification effects, whereas porosity, which is the vertical projection of the plant community structure, had a positive correlation with cooling and a negative correlation with humidification. Additionally, they noted that different vegetation types had distinct effects on cooling and humidification [39]. Increased LAI increases water demand and transmission power, whereas an increase in the number of pores directly corresponds to an increase in the number of water transport channels [40]. In addition, leaf shading and vertical diffusion of leaf deformation are reported to have a profound effect on atmospheric chemistry not only on temperature but also on the Earth’s surface and the atmospheric boundary layer [41].

The further into the forest, the lower the temperature at the highest temperature, and the higher the temperature at the lowest temperature. Compared to the lawn and Ti, the temperature difference is relatively low in larger UFs (Fig 2). The temperature reduction effect has a greater correlation with the highest temperature [42], and the temperature rises from the lowest average temperature to the inside of the forest because the temperature fluctuation is not large as the plants themselves act as an insulator [43]. The heat under the tree crown remains in the boundary layer due to the large surface area of the broad leaves. In contrast, the temperature seems to drop further in the coniferous forest with thin leaves because the air flow is disturbed [43]. In broadleaf forests, after November, as the leaves fall, the shade film that blocks the sun’s heat disappears, and the sunlight directly hits the tree trunks. This appears to be different from the phenomenon observed in coniferous forests. Temperature is lower from the forest boundary to the inside, indicating that the homeostasis (temperature resistance) essential for the survival of living things in the forest is stable. This variability differs from the research findings that the indoor temperature in summer is lower than that outside the forest, and the temperature in the forest during winter is higher than that outside the forest [44].

According to Sung et al [45], wind corridor forests are urban forests that allow the flow of cool and fresh air into urban areas. This improves air pollution and thermal insulation conditions in urban areas with poor climate conditions by introducing cool and fresh air (’kaltluft’ in German) from night forests to urban ventilation passages [46–48]. Wind corridor forests are classified into three divisions according to their role [45]: wind-generating, wind-connecting, and wind-spreading forests. Through wind corridor forests, the green area where the cold wind is generated and the linear green area are connected, and the cold wind moves to the city center. In this study, we found that the temperature from UFs continued to decrease in all seasons (Fig 4). It is deduced that the cold air generated from Mt. Cheonjang located in the center of the city will move to the city center (Ti) through the park area and linear green areas (roadside trees) (Fig 6). As for the temperature of the UF, Edmondson [49] found that the mean daily soil surface temperature over 11 months increased by 0.6°C over 5 km from the outside of the city to the center.

UFs are known to contribute to the urban temperature reduction effect via three processes: 1) shade effect, 2) evapotranspiration effect, and 3) by reflecting more solar radiation. This study supports the findings that woody vegetation in urban green spaces can cool the urban environment and have the potential to mitigate the UHI effect. The cooling effect of UF was determined through diurnal and seasonal changes, and the differences in the temperature reduction effect according to the forest type was also identified. Although this effect is perennial, it is particularly consistent with the onset of heat waves and heat illness, the two greatest potential risks associated with UHI, and is expected to grow in importance as the frequency of extreme weather events increases in response to climate change. The temperature reduction effect not only reduces the temperature in the forest; UFs over a certain size can also affect the temperature in the nearby downtown area. Further, small UFs are effective in reducing temperature and can serve as shelter for citizens from heat waves that are expected to occur periodically. Owing to the increase in UHI, it is necessary to consider not only the quantitative implementation of UFs in the city, but also the connection between various UF areas.

Recently, several researchers have published studies on urban/park cool islands (UCIs/PCIs). This refers to the phenomenon where green spaces and their surrounding environment have a lower temperature than the impervious surface [50, 51]. Additionally, recent studies have been conducted using remote-sensing technologies, such as MODIS, Landsat, and airborne data, to identify characteristics of urban green spaces, including cooling intensity, temperature threshold, and maximum cooling extent [52–54]. UCIs/PCIs are also important for human thermal comfort (HTC) by reducing the urban mean radiant temperature; thus, many studies have been conducted on the quantitative evaluation of the contribution of urban greening to HTC and thermal stress reduction [55–57]. Similar to the results of this study, the cooling effect of green spaces were found to be significant despite differences in climate and physical characteristics. Based on the results of this study, practical research should be conducted on the effect of reducing the apparent temperature directly felt by people using UFs of various shapes and sizes. In addition, future research on UFs should focus on the morphological and compositional aspects of cities.

Studies on UF temperatures currently focus on extreme conditions, such as heatwaves and cold spells that occur during the peak of summer and winter, respectively [27, 58–60]. In addition, research on the temperature reduction effects of UFs using remote sensing techniques is actively being conducted [61–63]. Analysis of temperature reduction effects using remote sensing techniques has the advantage of the capability to compare large spatial scales simultaneously. However, the use of remote sensing for temperature analysis is difficult due to instabilities in the data caused by atmospheric conditions (clouds); additionally, the predetermined imaging cycle and timing complicate obtaining data for desired times. Moreover, land surface temperature is used, resulting in limitations in obtaining information on the temperature below the tree canopy. Thus, to understand changes in the thermal environment in UFs according to their phenological characteristics, air temperatures must be continuously measured for more than one year [64]. Therefore, in this study, we confirmed the thermal environmental changes in UFs utilizing stable air temperature data collected at 30-minute intervals from Dec. 1. 2019–Nov. 30. 2020. Furthermore, we conducted research on the temperature reduction effects via UFs across various time scales, such as daytime and nighttime, seasonal, and monthly, and distinguished between various forest types (coniferous, deciduous, mixed forest, lawn, and urban park). These results can provide an important resource for understanding the physiological and environmental characteristics of urban forests for future climate regulation.

Since UFs provide various ESS to people living in the city, various considerations are needed for its creation plan. Nature-based solutions (NbS) promote UF networks as they have significant potential to reduce vulnerability to climate change and improve urban resilience [65]. In other words, NbS promote the maintenance, enhancement, and restoration of biodiversity and ecosystems to simultaneously solve several problems [12, 66]. Consideration of NbS will not only strengthen the network in the city center by accurately understanding the scale and functions of various UFs in the city, but also increase the connectivity between the inside and outside of the city [67]. The results of this study will help to create conflict-free landscape solutions for residents and the environment based on a clear understanding of urban space, provision, and demand for urban ESS.

## Materials and methods

### Study area

Korea is located in a mild climate zone at mid-latitudes, and Seoul is located in the center of the Korean Peninsula, surrounded by sub-urban regions. The average annual temperature of Seoul (1991–2020) is 12.8°C. The coldest month is January at −1.9°C, and the hottest month is August at 26.1°C, with an annual temperature difference of 28.0°C. The average annual precipitation is 1,417.9 mm, and the sum of precipitation in summer (June, July, August) is 892.1 mm, accounting for about 63% of the annual precipitation. On the other hand, the sum of precipitation in winter (December, January, February) is about 5% of the annual precipitation (www.weather.go.kr). In the central region of the Korean Peninsula, where the study was conducted, the record rainy season continued for 54 days from June 24 to August 16, 2020. High temperatures were recorded earlier than normal in June, and the unusually low temperature and heavy rain fell in July and August. There was a severe temperature change due to the heat wave that came after mid-August. That is, higher than normal temperatures were observed in June and August, and lower than normal temperatures were observed in July.

In this study, the Hongneung Experimental Forest and its surrounding regions, located east of Seoul, Korea, were selected as the target survey sites. Cheonjangsan Mountain, located at the Hongneung Experimental Forest, is approximately 140 m above sea level and is an important green space and UF in Dongdaemun-gu, which is densely populated and lacks park areas. The forest is composed mainly of trees and is systematically managed, making it easy to monitor and analyze temperature changes according to the type of UF and measure the cold air radiated into the city by the geographical characteristics of mountains at night. The Cheongnyangni area, about 1.5–2 km away from Cheonjangsan Mountain, is known as a representative urban area in northeastern Seoul due to its large floating population, vehicular traffic, and railway lines.

The measurement points were a total of 17 points, including 15 points in the Hongneung Experimental Forest and Cheonjangsan Mountain (7 coniferous forests, 5 broadleaved forests, 2 mixed forests, 1 lawn), one in the urban park (Yeonghuiwon), and one at a Ti near Cheongnyangni Station. The urban park is located on a straight line between the Hongneung experimental forest and the Ti. Data obtained from each of the different types of forest were compared with those of the urban park, lawn, and Ti. In addition, to compare and analyze the temperature difference between the UF and the residential area, data closest to the research site provided by the AWS of the Seoul Metropolitan Government were used.

### Field measurement

In this study, the temperature and humidity changes in the UFs were measured and analyzed using temperature and humidity sensors (SP-2000-20R, Vaisala, Finland). All sensors were adjusted for human height, approximately 1 m. The operating range of the sensor is from −35°C to 85°C. The temperature sensor accuracy is ±0.15°C (at a temperature range of −25 to 70°C) and ±0.10°C (at a temperature range of 20–30°C). The accuracy of the relative humidity sensor is ±2% (at a range of −20 to 70°C) and ±1% (at a range of 20–30°C). The temperature and relative humidity were automatically and continuously monitored at 30-min intervals from Dec. 1. 2019–Nov. 30. 2020. The measured data were re-classified into 1-hour units and then analyzed. In the summer, when there are more hot days and tropical nights, the analysis was conducted after excluding days when rainfall was recorded in AWS.

To identify the species and forest structures of the 16 locations, excluding the lawn where no trees existed, a forest inventory was conducted. The survey was conducted using 10×10 m (0.01 ha) square plots centered on the locations of the weather sensors, and all trees with a diameter at breast height (DBH) of 6 cm or more were measured [68]. Trees that forked below breast height were considered individual trees, and their information was measured separately. Ten parameters were investigated, namely species, DBH, basal area, height, stem volume, crown depth, clear length, crown diameter, crown projection area, and crown volume. The investigation was performed during the leaf-out period (April to May) of deciduous trees, when tree heights and dead branches could be clearly identified in densely forested areas.

### Data analysis

The difference between temperatures measured at each UF point and the temperature data obtained from the AWS of the residential area was considered as a cooling effect (Table 2). The lower the calculated value here, the cooler the gray infrastructure. The measured data were divided into diurnal, monthly, and seasonal, and the effect of temperature reduction at each UF point was compared and analyzed. Based on the average sunrise (06:26) and sunset (18:37) time data provided by the Korea Astronomy and Space Science Institute (astro.kasi.re.kr), we defined daytime as 06:00–19:00 and nighttime as 20:00–05:00. This was done to obtain the same number of samples during each time period for the comparison of temperature differences between day and night during different seasons [69, 70]. The criteria for the classification of hot days and tropical nights were set as a daily maximum temperature of 33°C or higher and a daily minimum temperature of 25°C or higher, respectively. Data preprocessing, data comparison, and statistical analysis were conducted using R (version 3.0.2).

**Table 2:** Formulas for calculating the cooling effect (temperature difference) of urban forests.

Name	Formula
*T_c_*	*T_Con_*–*T_AWS_*
*T_b_*	*T_Bit_*–*T_AWS_*
*T_m_*	*T_Mix_*–*T_AWS_*
*T_l_*	*T_Lawn_*–*T_AWS_*
*T_p_*	*T_Park_*–*T_AWS_*
*T_t_*	*T_Ti_*–*T_AWS_*

AWS: Automatic Weather System; Con: coniferous forest; Blt: broadleaved forest; Mix: mixed forest; Lawn: lawn land; Park: urban park; Ti: traffic island.
